# Rapidly evolving changes and gene loss associated with host switching in *Corynebacterium pseudotuberculosis*

**DOI:** 10.1371/journal.pone.0207304

**Published:** 2018-11-12

**Authors:** Marcus Vinicius Canário Viana, Arne Sahm, Aristóteles Góes Neto, Henrique Cesar Pereira Figueiredo, Alice Rebecca Wattam, Vasco Azevedo

**Affiliations:** 1 Department of General Biology, Universidade Federal de Minas Gerais, Belo Horizonte, Minas Gerais, Brazil; 2 Leibniz Institute on Aging, Fritz Lipmann Institute, Jena, Germany; 3 Department of Microbiology, Universidade Federal de Minas Gerais, Belo Horizonte, Minas Gerais, Brazil; 4 AQUACEN, National Reference Laboratory for Aquatic Animal Diseases, Ministry of Fisheries and Aquaculture, Universidade Federal de Minas Gerais, Belo Horizonte, Minas Gerais, Brazil; 5 Biocomplexity Institute of Virginia Tech, Virginia Tech, Blacksburg, Virginia, United States of America; Academia Sinica, TAIWAN

## Abstract

Phylogenomics and genome scale positive selection analyses were performed on 29 *Corynebacterium pseudotuberculosis* genomes that were isolated from different hosts, including representatives of the Ovis and Equi biovars. A total of 27 genes were identified as undergoing adaptive changes. An analysis of the clades within this species and these biovars, the genes specific to each branch, and the genes responding to selective pressure show clear differences, indicating that adaptation and specialization is occurring in different clades. These changes are often correlated with the isolation host but could indicate responses to some undetermined factor in the respective niches. The fact that some of these more-rapidly evolving genes have homology to known virulence factors, antimicrobial resistance genes and drug targets shows that this type of analysis could be used to identify novel targets, and that these could be used as a way to control this pathogen.

## Introduction

Population genetics and genomic approaches increase our understanding of both natural selection and molecular evolution. Alleles with adaptive mutations increase in frequency in what is known as positive selection, and these mutations have been identified by comparing nucleotide sequences between different populations [1–3]. Codon substitution models, which compare a non-synonymous (*d*_*N*_) to synonymous (*d*_*S*_) substitution rate (as ω = *d*_*N*_ / *d*_*S*_), can be used to determine if the mutations that change the amino acid (*d*_*N*_) in a specific position are adaptive (ω > 1, positive selection), deleterious (ω < 1, negative selection) or neutral (ω = 1, neutral evolution) [4]. Research has shifted from looking at selective pressures on individual genes to a broad examination that looks for genes under selective pressure across entire genomes [5–7], and the pipelines developed to examine this often involve orthologous group identification, codon based alignments, phylogenetic tree reconstruction, and models of codon evolution [3,8].

The interactions between a host and its infecting pathogen have been of particular interest to those interested in positive selection, particularly in the interactions that involve the immune and defense mechanisms deployed by the host. Pathogen genes that have been identified as being under positive selection have been found to be involved in regulation, modulation and modification of the host immune response, membrane lipid metabolism, certain cell wall processes, and receptor mediated binding [6,9], all of which could play a role in host-pathogen interactions. Several studies have examined selective pressures and the response in many important pathogenic bacteria, including *Escherichia coli* [9,10], *Salmonella* [10], *Staphylococcus aureus* [11], *Mycobacterium tuberculosis* [12–14], *Shigella flexneri* [9], and members of the *Streptoccocus* [15], *Campylobacter* [16] and *Leptospira* [17,18] genera.

*Corynebacterium pseudotuberculosis* is a Gram-positive, pleomorphic and facultative intracellular bacterium of veterinary and medical relevance. It has a global distribution [19], and it causes economic losses in animal production. Control methods, such as diagnosis, vaccines and antibiotics remain elusive [19]. It is separated into two biovars based on host preference and nitrate reduction. Biovar Ovis (nitrate negative) is the causative agent of Caseous Lymphadenitis (CLA), a chronic disease in goats and sheep [20,21]. Ovis has also been isolated from cattle [22], camels [23], and humans [21,24,25], causing skin lesions or lymphadenitis. Isolates from the Equi biovar (nitrate positive) are known for causing Oedematous Skin Disease (OSD) in buffaloes [26]. Equi isolates have also been found in horses [27,28], cattle [22,29] and camels [30], with different manifestation in each host species. Cattle and camels are the only cross-over hosts in that both Ovis and Equi strains have been isolated from them, but each biovars present a different disease phenotype. Ovis has never been found in horses or buffalo, and no sheep or goats have been found to be infected by any strain belonging to the Equi biovar. However, an experimental infection of a strain isolated from a buffalo and part of the Equi biovar caused CLA in sheep [31].

While previous work has identified changes specific to each of the *C*. *pseudotuberculosis* biovars [32,33], no one has been able to identify any genes that are involved in the interactions between pathogen and host species. A single exception is probably the presence of a particular prophage that harbors the diphtheria toxin (DT) and is found only in strains isolated from buffalo (Equi biovar) [33]. Phylogeny of the species show that the two biovars are clearly distinct. In this work, we examined nucleotide changes in genes shared by both biovars in order to identify differences in selective pressure as a means to explore the evolution of this pathogen, and to distinguish genes that might be involved in host-pathogen interactions and host preference.

## Materials and methods

### Genomes and reannotation

Positive selection analysis that includes all of the genes in a single genome, and then compares a group genomes, is computationally expensive [5,6]. We limited the sampling to 29 complete genomes of *C*. *pseudotuberculosis* that were retrieved from GenBank. These genomes represent isolates from both biovars, and from each type of host that has been found to be infected with *C*. *pseudotuberculosis*. A maximum of five genomes were included from each type of host, depending upon availability (Table 1). All genomes were all consistently annotated using the RASTtk (Rapid Annotation Using Subsystem Technology) [34] annotation service in the Pathosystem Resource Integration Center (PATRIC) [35].

**Table 1:** *Corynebacterium pseudotuberculosis* genomes used in positive selection analysis.

Strain	Biovar	Host	Country	Access no
E56	Ovis	Sheep	Egypt	CP013699.1
PA01	Ovis	Sheep	Brazil	CP013327.1
C231	Ovis	Sheep	Australia	CP001829.1
MEX25	Ovis	Sheep	Mexico	CP013697.1
N1	Ovis	Sheep	Equatorial Guinea	CP013146.1
1002B	Ovis	Goat	Brazil	CP012837.1
VD57	Ovis	Goat	Brazil	CP009927.1
PO222/4-1	Ovis	Goat	Portugal	CP013698.1
MEX1	Ovis	Goat	Mexico	CP017711.1
MEX9	Ovis	Goat	Mexico	CP014543.1
P54B96	Ovis	Wildebeest	South Africa	CP003385.1
267	Ovis	Llama	USA	CP003407.1
48252	Ovis	Human	Norway	CP008922.1
FRC41	Ovis	Human	France	CP002097.1
I19	Ovis	Cow	Israel	CP002251.1
29156	Ovis	Cow	Israel	CP010795.1
262	Equi	Cow	Belgium	CP012022.1
I37	Equi	Cow	Israel	CP017384.1
162	Equi	Camel	UK	CP013260.1
258	Equi	Horse	Belgium	CP003540.2
MB14	Equi	Horse	USA	CP013261.1
E19	Equi	Horse	Chile	CP003540.2
MEX30	Equi	Horse	Mexico	CP017291.1
CIP52.97	Equi	Horse	Kenya	CP003061.2
31	Equi	Buffalo	Egypt	CP003421.3
32	Equi	Buffalo	Egypt	CP015183.1
33	Equi	Buffalo	Egypt	CP015184.1
36	Equi	Buffalo	Egypt	CP015186.1
48	Equi	Buffalo	Egypt	CP015191.1

### Genome scale positive selection analysis

Positive selection analysis using branch-site models has been used to identify genes and specific codons (sites) that are under positive selection in specific phylogenetic lineages (also called directional selection. When doing this type of comparison, the lineage to be tested for positive selection is identified as the “foreground”, and the genomes compared to that foreground lineage are labeled as “background”. This comparison will identify specific sites that are under positive selection (ω > 1) only in the foreground lineage, evidencing its adaptive mutations [36,37]. Once identified, the functional roles of these genes can be explored, and they can play in part in future hypothesis generation [38].

The PosiGene pipeline [7] was used to perform genome-scale positive selection in this analysis using branch-site models. Multifasta files containing the protein-coding sequences of each gene of the 29 genomes were generated, with the RASTtk sequence IDs modified to a format suitable for PosiGene (RASTtk-based IDs) using a modified version of the script extract_aa_nt_from_gb.pl (S1 File) [6]. The input files for each genome are provided in S2 File.

#### Ortholog group assignment

The PosiGene module “create_catalog”, which uses a BLASTp best-bidirectional hit analysis [39,40], was used to assign ortholog groups. Each group was named after the ID from the sequence of a reference genome and only ortholog groups that have a sequence from an anchor genome were analyzed. A reference or anchor genome was selected according to the biovar of the foreground genomes, to avoid missing genes that are more common in a specific biovar. Strain 31, a buffalo isolate, was selected as the reference and anchor genome for Equi biovar, and strain 1002B (goat) was selected for Ovis.

#### Alignments, gene trees and species tree

The PosiGene module “alignments” was used to generate multiple sequence alignments. This module also created a phylogenetic tree for each ortholog group, and a species tree (consensus tree). The species tree was used for realignment of the nucleotide sequences by codon and posterior identification of the target groups.

A sequence filter based on similarity, with a minimal sequence identity of 50%, was performed to ensure the analysis of one sequence per genome on each ortholog group [7]. For each gene sequence from the reference genome, the orthologs from all genomes were assigned by progressive protein alignments using CLUSTALW [41,42].

A phylogenetic tree of each ortholog group was generated by alignment filtering using GBLOCKS [43] and phylogenetic reconstruction by the parsimony method and jackknifing using DNAPARS from the PHYLIP package [44]. For the species tree, a consensus tree was calculated using PHYLIP’s CONSENSE program. Codon level alignments were generated using PRANK [45] for each ortholog group that had at least three sequences, and also for the species tree.

#### Target groups

**Fig 1:**
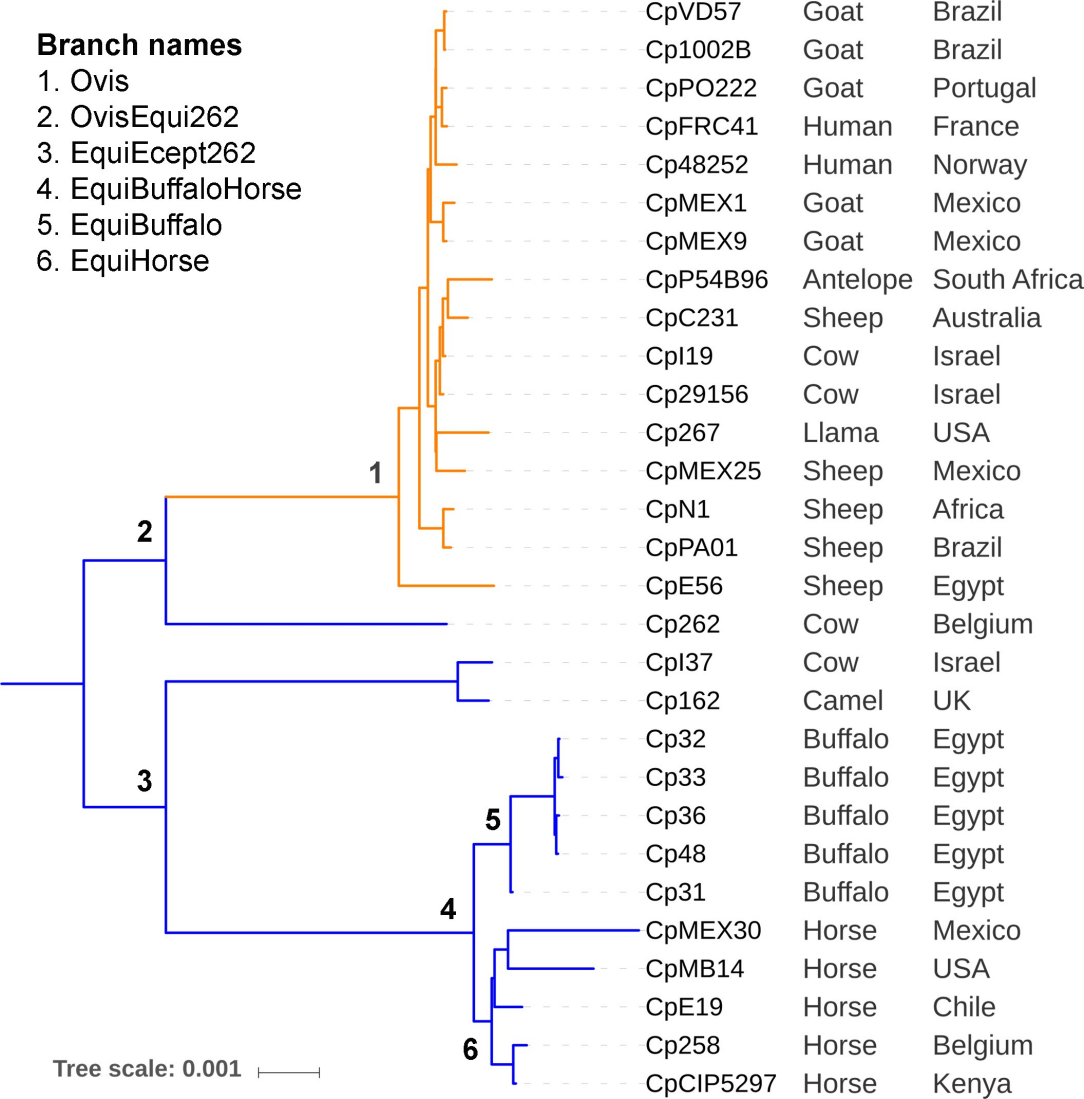
Target groups (foreground branches) 1 to 6 of a *Corynebacterium pseudotuberculosis* phylogeny.

The species tree had to be manually rooted prior to the selection target groups. To identify the most ancestral branch of *C*. *pseudotuberculosis*, a second tree was generated that included *C*. *ulcerans* strain 210932 (CP009500.1) [46] to root the tree (S1 Fig) and identify the most ancestral *C*. *pseudotuberculosis* clade. The first *C*. *pseudotuberculosis* species tree (without *C*. *ulcerans*) was then manually rooted using MEGA 7 [47] and visualized with iTOL 4.2.3 (itol.embl.de) (Fig 1) to identify foreground groups and to be used in the next step of the PosiGene pipeline. We compared this tree (Fig 1) with trees generated by other two methods to compare and confirm phylogenetic placement. One of these comparison trees was built using the PEPR (https://github.com/enordber/pepr.git) (S2 Fig), a pipeline that uses the core proteome and builds an alignment of all the genes shared across all genomes. Another comparison tree was built using MEGA 7 and the Maximum Likelihood method [48]. This tree was generated based on the alignment the *rpoB* gene (S3 Fig), which has been described a good discriminator for differentiating between *Corynebacterium* species [49].

**Fig 2:**
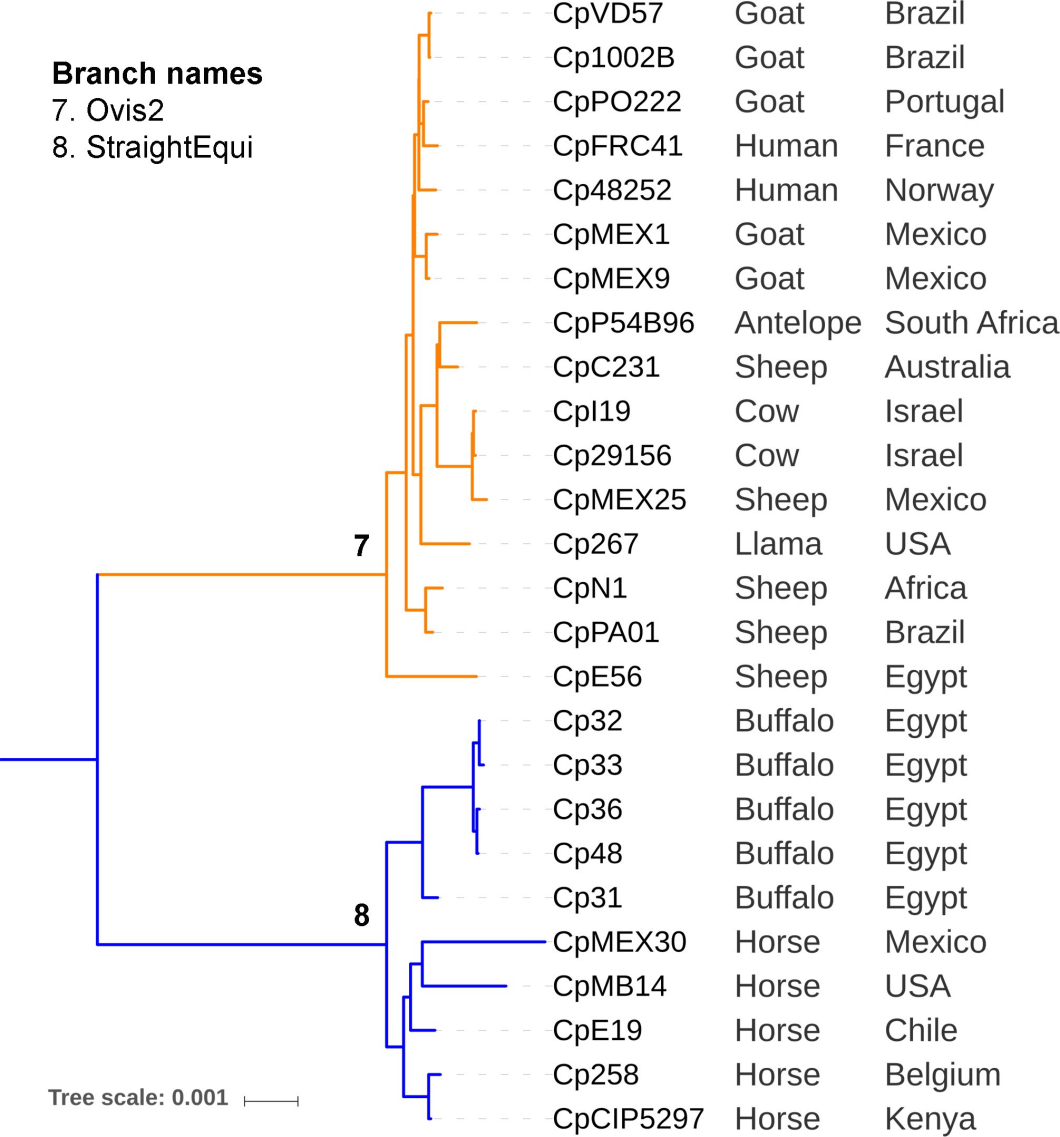
Target groups (foreground branches) 7 and 8 of a *Corynebacterium pseudotuberculosis* phylogeny excluding the Equi strains 262, I37 and 162.

Eight separate foreground groups were used as input for PosiGene. These were selected based on the clades that were identified by the phylogenetic trees (Figs 1 and 2). This resulted in eight separate analyses, each one comparing a foreground group with the remaining groups in the tree (background), to identify adaptive mutations that occurred only in the last common ancestor of the foreground group. The target groups are listed in Table 2 and are represented in the phylogenomic trees of Figs 1 and 2.

**Table 2:** Groups of foreground and background lineages of *Corynebacterium pseudotuberculosis* analyzed by branch-site models.

Group number	Group name	Foreground (genomes)	Background (genomes)	Reference/anchor genome
**1**	Ovis	All Ovis genomes (16)	All Equi genomes (13)	Cp1002B (Ovis)
**2**	OvisEqui262	All Ovis genomes and Equi 262 (17)	All Equi genomes except 262 (12)	Cp1002B (Ovis)
**3**	EquiExcept262	All Equi genomes except 262 (12)	All Ovis genomes and Equi 262 (17)	Cp31 (Equi)
**4**	EquiBuffaloHorse	Equi genomes from buffalo and horse only (10)	All other Ovis and Equi genomes (19)	Cp31 (Equi)
**5**	EquiBuffalo	Equi genomes from buffalo only (5)	All other Equi and Ovis genomes (24)	Cp31 (Equi)
**6**	EquiHorse	Equi genomes from horse only (5)	All other Equi and Ovis genomes (25)	Cp31 (Equi)
**7**	Ovis2	All Ovis genomes (16)	Equi genomes from buffalo and horse only (10)	Cp1002B (Ovis)
**8**	StraightEqui	Equi genomes from buffalo and horse only (10)	All Ovis genomes (16)	Cp31 (Equi)

#### Positive selection module

The codeml program of the PAML package [8] was used to identify sites under positive selection by a branch-site test [36,37], which uses each gene sequence alignment and its phylogenetic gene tree as input. The likelihood ratio test (LRT) calculates and compares the likelihood of a null model, where all sites are considered to evolve under neutral (ω = 1) or negative selection (ω < 1), and an alternative model that assumes that the same sites are under positive selection (ω > 1) on the foreground branch only. The *p*-value for the LRT is calculated via a χ^2^ distribution, with one degree of freedom. For each site with a significant *p-value*, the Bayes empirical Bayes (BEB) method was used to calculate the posterior probability [50]. In addition to the *p*-value, the PosiGene pipeline provides the significance value for the Bonferroni correction and Benjamini–Hochberg false discovery rate (FDR) [51]. We considered positive selection when *p* < 0.05 for FDR only, as Bonferroni is too conservative and can lead to many false negatives [52]. For each gene that was identified as being under positive selected, the sequence alignment was tested for evidence of intragenic recombination, as it can lead to an alignment of non-homologous codons and possible false positive results [53,54]. As no single method performs optimally under all scenarios, our strategy involved a combination of all of them [55]. We used PhiPack [56] to test for evidence of recombination using the methods Pairwise Homoplasy Index (PHI) [56], Neighbor Similarity Score (NSS) [57] and Maximum Chi-Square [58]. We considered recombination when *q* < 0.05 for PHI and at least one another test [6].

### Gene functional characterization and location

**Fig 3:**
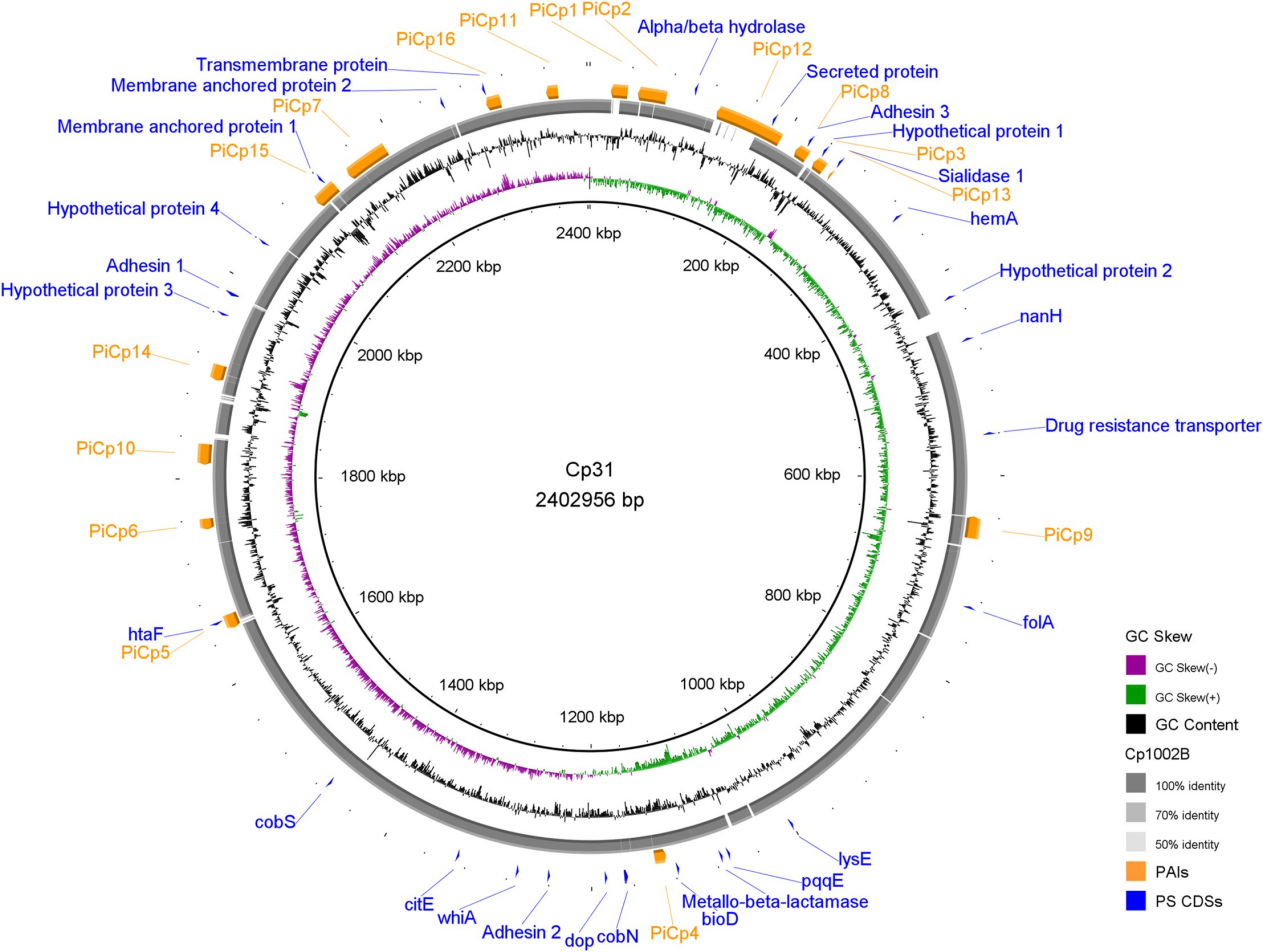
Circular map showing the position of pathogenicity islands and positively selected genes in relation to *Corynebacterium pseudotuberculosis* strain 31 genome. PAI–Pathogenicity Island, PS–positively selected, CDS–coding sequences.

For each gene that the pipeline identified as being under positive selection, the sequence from the anchor genome was checked for the presence of functional domains using the InterProScan Database (https://www.ebi.ac.uk/interpro/search/sequence-search), and for metabolic activity using PATRIC’s Pathway Summary [35]. PATRIC’s Protein Family Sorter was used to verify the distribution of specific genes across the genomes. GIPSy [59] was used to verify the location of positively selected genes in relation to 16 pathogenicity islands that have been previously described [32], using *C*. *glutamicum* ATCC1302 (NC_006958.1) as the non-pathogenic reference. The positions of the positively selected genes were plotted in a circular map generated using BRIG Fig 3 [60].

## Results and discussion

We used genome-scale positive selection analyses to identify adaptive mutations in specific lineages (branches or foregrounds) of *C*. *pseudotuberculosis*, and explored differences that could be correlated with biovar and isolation host.

### Positively selected genes

The complete results for positive selection analysis for each foreground are provided (S3 File), as are the GenBank and RASTtk locus tags for each gene (S1 Table). Twenty-seven genes were identified as being under positive selection (Table 3) and the number of positively selected sites for each foreground is given in Table 4. Seven of the eight foreground groups had genes that were identified as being under positive selection, with the sole exception being Branch 6 (EquiHorse, Table 2). None of these 27 genes were significant for the recombination detection method (S2 Table).

**Table 3:** List of positively selected genes in *Corynebacterium pseudotuberculosis* in different branches (FDR < 0.05).

GenBank ID (Equi/ Ovis)	1	2	3	4	5	7	8	Product (Gene)	Function	PAI	Reference (Drug target or Vaccine)
Cp31_0488/ Cp1002B_0499	X							Drug resistance transporter	Resistance	-	[62,63]
Cp31_1168/ Cp1002B_1500	X							Citrate lyase beta chain (*citE*)	Metabolism	-	[64]
Cp31_1468/ Cp1002B_1186	X							Cell-surface hemin receptor (*hatF*)	Transport	PiCp5	-
Cp31_2169/ Cp1002B_0189	X							Hypothetical protein 1 (no domains)	Unknown	PiCp3	-
Cp31_0206/ Cp1002B_0207		X	X					Sialidase 1	Virulence	PiCp13	[65,66]
Cp31_0638/ Cp1002B_2037		X						Dihydrofolate reductase (*folA*)	Metabolism	-	[67]
Cp31_0945/ Cp1002B_1731		X						Coenzyme PQQ biosynthesis protein E (*pqqE*)	Metabolism	-	-
Cp31_0950/ Cp1002B_1726		X						Metallo-beta-lactamase	Resistance	-	[68]
Cp31_0985/ Cp1002B_1689		X						Dethiobiotin synthetase (*bioD*)	Metabolism	-	[69,70]
Cp31_1044/ Cp1002B_1624		X						Pup deaminase (*dop*)	Metabolism	-	[71,72]
Cp31_1309/ Cp1002B_1363		X	X			X	X	Cobalt chelatase subunit CobS (*cobS*)	Metabolism	-	-
Cp31_1724/ Cp1002B_0908		X	X			X	X	Hypothetical protein 3 (no domains)	Unknown	-	-
Cp31_1868/ Cp1002B_0763		X						Membrane anchored protein 1	Unknown	PiCp13	-
Cp31_0109/ Cp1002B_0104			X					Alpha / beta hydrolase	Unknown	-	[73]
Cp31_2015/ Cp1002B_2083			X					Transmembrane protein	Unknown	PiCp16	-
Cp31_2279/ -			X	X				Adhesin 1 (membrane anchored)	Adhesion	-	-
Cp31_0366/ Cp1002B_0381				X				Hypothetical protein 2 (no domains)	Unknown	-	-
Cp31_1094/ Cp1002B_1575				X				Adhesin 2 (membrane anchored)	Adhesion	-	-
Cp31_1977/ Cp1002B_0655				X				Membrane anchored protein 2	Unknown	-	-
Cp31_0279/ Cp1002B_0289					X			Glutamyl-tRNA reductase (*hemA*)	Metabolism	-	[74]
Cp31_1028/ Cp1002B_1640					X			Cobaltochelatase subunit CobN (*cobN*)	Metabolism	-	-
Cp31_1117/ Cp1002B_1551					X			Sporulation regulator WhiA-like (*whiA*)	Cell division	-	-
Cp31_0142/ Cp1002B_0139						X	X	Secreted protein	Unknown	PiCp12	-
Cp31_0180/ Cp1002B_0178						X	X	Adhesin 3 (thioester domain)	Adhesion	PiCp8	-
Cp31_0399/ Cp1002B_0408						X	X	Sialidase 2 (*nanH*)	Metabolism	-	[65,66]
Cp31_0893/ Cp1002B_1784						X	X	Lysine exporter protein (*lysE*)	Transport	-	[75]
Cp31_2281/ Cp1002B_0835						X		Hypothetical protein 4 (no domains)	Unknown	-	

PAI–Pathogenicity island

**Table 4:** Number and percentage of positively selected sites in *Corynebacterium pseudotuberculosis*.

GenBank ID (Equi/ Ovis)	Alignment	Positively selected sizes per foreground (%)							Product (Gene)
		1	2	3	4	5	7	8	
Cp31_0488/ Cp1002B_0499	473	1 (0.21)	-	-	-	-	-	-	Drug resistance transporter
Cp31_1168/ Cp1002B_1500	300	2 (0.67)	-	-	-	-	-	-	Citrate lyase beta chain (*citE*)
Cp31_1468/ Cp1002B_1186	721	1 (0.14)	-	-	-	-	-	-	Cell-surface hemin receptor (*hatF*)
Cp31_2169/ Cp1002B_0189	208	13 (6.25)	-	-	-	-	-	-	Hypothetical protein 1 (no domains)
Cp31_0206/ Cp1002B_0207	465	-	5 (1.08)	5 (1.08)	-	-	-	-	Sialidase 1
Cp31_0638/ Cp1002B_2037	175	-	1 (0.57)	-	-	-	-	-	Dihydrofolate reductase (*folA*)
Cp31_0945/ Cp1002B_1731	412	-	1 (0.24)	-	-	-	-	-	Coenzyme PQQ biosynthesis protein E (*pqqE*)
Cp31_0950/ Cp1002B_1726	201	-	2 (1.00)	-	-	-	-	-	Metallo-beta-lactamase
Cp31_0985/ Cp1002B_1689	229	-	1 (0.44)	-	-	-	-	-	Dethiobiotin synthetase (*bioD*)
Cp31_1044/ Cp1002B_1624	510	-	3 (0.59)	-	-	-	-	-	Pup deaminase (*dop*)
Cp31_1309/ Cp1002B_1363	360	-	3 (0.83)	3 (0.83)	-	-	4 (1.11)	4 (1.11)	Cobalt chelatase subunit CobS (*cobS*)
Cp31_1724/ Cp1002B_0908	42	-	1 (2.38)	1 (2.38)	-	-	1 (2.38)	1 (2.38)	Hypothetical protein 3 (no domains)
Cp31_1868/ Cp1002B_0763	297	-	10 (3.37)	-	-	-	-	-	Membrane anchored protein 1
Cp31_0109/ Cp1002B_0104	286	-	-	2 (0.7)	-	-	-	-	Alpha / beta hydrolase
Cp31_2015/ Cp1002B_2083	347	-	-	3 (0.86)	-	-	-	-	Transmembrane protein
Cp31_2279/ -	868	-	-	20 (2.3)	23 (2.65)	-	-	-	Adhesin 1 (membrane anchored)
Cp31_0366/ Cp1002B_0381	44	-	-	-	2 (4.55)	-	-	-	Hypothetical protein 2 (no domains)
Cp31_1094/ Cp1002B_1575	604	-	-	-	14 (2.32)	-	-	-	Adhesin 2 (membrane anchored)
Cp31_1977/ Cp1002B_0655	298	-	-	-	6 (2.01)	-	-	-	Membrane anchored protein 2
Cp31_0279/ Cp1002B_0289	432	-	-	-	-	1 (0.23)	-	-	Glutamyl-tRNA reductase (*hemA*)
Cp31_1028/ Cp1002B_1640	1201	-	-	-	-	2 (0.17)	-	-	Cobaltochelatase subunit CobN (*cobN*)
Cp31_1117/ Cp1002B_1551	329	-	-	-	-	1 (0.30)	-	-	Sporulation regulator WhiA-like (*whiA*)
Cp31_0142/ Cp1002B_0139	213	-	-	-	-	-	15 (7.04)	15 (7.04)	Secreted protein
Cp31_0180/ Cp1002B_0178	518	-	-	-	-	-	31 (5.98)	31 (5.98)	Adhesin 3 (thioester domain)
Cp31_0399/ Cp1002B_0408	680	-	-	-	-	-	92 (13.53)	92 (13.53)	Sialidase 2 (*nanH*)
Cp31_0893/ Cp1002B_1784	243	-	-	-	-	-	3 (1.23)	3 (1.23)	Lysine exporter protein (*lysE*)
Cp31_2281/ Cp1002B_0835	60	-	-	-	-	-	10 (16.67)	-	Hypothetical protein 4 (no domains)

The branch-site models used in the analysis identify sites under positive selection only in the foreground group (branch). To confirm our results, we checked to see if the same sites identified as being under positive selected sites are also identified when a subset of the genomes that had been previously tested as foreground were used as the new foreground genomes (S4 Fig). In this case, the previously identified sites would not be expected to be identified with the new foreground genomes. The results show that none of the previously identified genes were positively selected within these genome subsets (S4 File), thus confirming the previous results (Table 3).

An analysis of the 27 genes identified as being under positive selection showed that they played a variety of functional roles that includes activity in metabolism, cell division, resistance, transport, adhesion, or were identified as hypothetical proteins with unknown functions. Many of these genes have previously been suggested as drug or vaccine targets (Tables 3 and 4). Seven of them are located in areas that have been previously identified as pathogenicity islands [32] (Fig 3, Table 3). The functional categories assigned to the genes in these islands have previously been included in a list of niche/virulence factors involved in pathogenesis for the *Corynebacterium* genus [61]. Some of the genes appeared to be exposed on the cell surface. Proteins located at the interface between bacteria and the environment are more likely to undergo positive selection [9], so it would not be surprising if some of the genes we detected (Table 3) play a role in the dynamics of the host-pathogen interaction. Some of the processes that had genes identified as being under positive selection include nutrient uptake, modulation of the host immune response, resistance and receptor-mediated binding [6,9] (Table 3). In those proteins, positive selection could act as a protective measure to avoid attachment by antibodies or phages, instead of a response related to the protein function [9].

### Positive selection in each target group

#### Adaptations in Ovis biovar (Foreground 1: Ovis)

Several studies have identified phenotypic and genotypic changes that differentiate the Ovis and Equi biovars. These include differences in nitrate reduction [76], changes in serotype and disease manifestation in the guinea pig model host [77], and pathogenicity islands that are biovar specific [32]. In addition, the Ovis clade has been documented as having a higher genomic similarity across its members than what is seen in Equi [32,33].

Our examination of the Ovis clade (Foreground 1, Fig 1) compared the genomes from 16 Ovis isolates to 13 from Equi, with Cp1002B selected as the anchor (Table 2). This comparison revealed adaptive mutations in four genes (Cp31_1168, Cp31_0488, Cp31_1468 and Cp31_2169) that have occurred in Ovis since it separated from Equi (Table 3), providing an indication of specific selective pressures imposed upon this group. Three of these specific genes (Cp31_1168, Cp31_0488, Cp31_1468) have defined functions, while the fourth (Cp31_2169) is annotated as a hypothetical protein. Two of the genes with described functions are involved in the use of carbon and iron sources (*citE*, Cp31_1168 and *htaF*, Cp31_1468), and the third is a drug transporter that is used in competition with other microorganisms (Drug transporter, Cp31_0488) (Table 3). Two of these genes, *citE* (Cp31_1168) [64] and the drug resistance transporter Cp13_0488 [62], are homologs to previously identified drug targets [62,64], and the hypothetical protein (Cp31_2169) is located in a pathogenicity island.

#### Adaptations shared by Ovis and Equi strain 262 (Foreground 2: OvisEqui262)

Phylogenetic analysis showed that Equi strain 262 is closer to the Ovis biovar than it is to the Equi (Fig 1). To identify probable adaptive mutations that Equi 262 and genomes in the Ovis biovar share that differentiates them from the broader Equi clade, we compared these 17 genomes to the remaining 12 Equi genomes, with Cp1002B once again used as the anchor (Table 2). The 262 genome and all of the 17 belonging to Ovis share nine genes that were identified as being under adaptive selection (Table 3). Two play a role in virulence or antimicrobial resistance, and five have well-established roles in metabolism (Table 3). Sialidases have been associated with virulence in *Corynebacterium* [65,78], and Cp31_0206 is the one of two genes in this group that is located in a known pathogenicity island. The role of beta lactamases in drug resistance is well known, and the gene with this functional description (Cp31_0950) appears to be experiencing selective pressure within this group. Other genes indicated in making adaptive changes play an important metabolic role (Cp31_0638, Cp31_0638, Cp31_0945, Cp31_0985, Cp31_1044 and Cp31_1309), while the functions of the membrane anchored protein (Cp31_1868) in PiCp13 and a hypothetical protein (Cp31_1724) are not yet known.

Several of these genes identified in this group have homology to genes that have previously been suggested as possible drug targets in *Mycobacterium tuberculosis*, which is part of the CNMR group that includes *Corynebacterium*. These include the sialidase [66], dethiobiotin synthetase (*bioD*) [69], dihydrofolate reductase (*folA*) [67], pup deamidase (*dop*) [71,72] and the metallo-beta-lactamase [68].

#### Adaptations in the monophyletic Equi clade (Foreground 3: EquiExcept262)

In this group, we searched for positive selection only within the monophyletic lineage of Equi, which includes twelve genomes that were isolated from a variety of large mammals (Table 1). Although Cp262 is part of the Equi biovar, it was not included in this particular analysis because our phylogenetic analysis showed that it is more closely aligned with the Ovis clade than with the other Equi genomes (Fig 1). This comparison is a reverse of the previous one, as it looks for adaptive changes in the 12 Equi genomes compared to the 17 genomes that include the single 262 Equi and the 16 Ovis isolates. Strain Cp31 was used as the anchor (Table 2). This comparison revealed six genes under positive, adaptive selection in the 12 Equi genomes, and the fact that they do not occur in the other genomes show that the changes occurred after divergence with the common ancestor these Equi genomes share with 262. These include genes related to nutrition and evasion of the host immune response (Sialidase 1, Cp31_0206), acetyl-CoA and DNA synthesis, fermentation (*cobS*, Cp31_1309), an adhesion (Adhesin 1, Cp31_2279), and three genes of undetermined function (Cp31_1724, Cp31_0109 and Cp31_2015). Several of these genes (Cp31_0109, Cp31_2015 and Cp31_2279) were only identified in this particular comparison, with Adhesin 1 (Cp31_2279) being perhaps the most interesting as these types of genes are known virulence factors. It has 20 sites under positive selection (Table 4 and S3 File). Other genes found to be under positive selection in this group include an alpha/beta hydrolase (Cp31_0109), a transmembrane protein (Cp31_2015), and a hypothetical protein (Cp31_1724), but the roles that these genes have in the interaction with the hosts they infect has yet to be determined.

#### Adaptations shared by strains isolated from buffalo and horse (Foreground 4: EquiBuffaloHorse)

An examination of the Equi clade (Foreground 3, Fig 1) shows two distinct sub-branches that separate Equi genomes isolated from a cow (CpI37) and a camel (Cp162) from those isolated from horses and buffalo (Foreground 4, Fig 1). To identify genes under positive selection in the genomes united by Foreground 4, we compared the 10 buffalo and horse isolates to all the other 19 genomes in the analysis, using Cp31 as the anchor (Table 2). This comparison revealed four genes under positive selection within these genomes isolated from horses and buffalo, which included known surface exposed proteins and a hypothetical protein. Positive selection was found in Adhesin 2 (Cp31_1094) and in the Equi exclusive Adhesin 1 (Cp31_2279, 23 sites). Seeing the adhesin genes responding to selective pressure in the Equi biovar indicates that these proteins play an important role in the particular niche these organisms inhabit. These differences could help the Equi isolates adapt to the different hosts that they are able to utilize, which presumably includes adhesion to specific cell receptors. Moreover, one of these adhesins (Cp31_2279) was also identified in the Branch 3 comparison mentioned above, indicating that this particular gene is responding uniquely to different selective pressures that are imposed on each of these clades.

#### Adaptations in strains isolated from buffalo (Foreground 5: EquiBuffalo) and horse (Branch 6: EquiHorse)

In a previous analysis, buffalo strains were shown to be clonal, with 94.7% shared genes in the core genome [33]. They compose a monophyletic cluster and they were seen to differ from the horse isolates mainly by an exclusive *tox*^+^ prophage [33]. Isolates from buffalo were the only *C*. *pseudotuberculosis* strains shown to produce diphtheria toxin [31,79–83]. This information supports the hypothesis where the presence of the prophage, specifically its diphtheria toxin (*tox*), is required for *C*. *pseudotuberculosis* to infect this buffalo, and this has been suggested as a potential vaccine target [33]. In contrast, the genomes isolated from horses only share 42.5% of their genes in a prior study and no genes related to the different disease phenotypes were found [84]. It is clear that one of the main differences between the horse and buffalo isolates are the presence of the prophage and the diphtheria toxin [33], which fits the “stable ecotype” model where adaptive genes allowed expansion into a new niche (the buffalo host), and then the founder mutant reproduces clonally [85].

We searched for positive selection in the Equi clades isolated from buffalo and horses separately (Foreground 5, Fig 1). We compared the 5 genomes isolated from buffalo to all other *C*. *psedotuberculosis* genomes used in the analysis, using the Cp31 genome as the anchor (Table 2). Three genes were found to be under positive selection only in these buffalo isolates, and they include genes *hemA* (Cp31_0279), *cobN* (Cp31_1028 are related to biosynthesis of cofactors used in important biological process, while *whiA* (Cp31_1117) is involved in cell division regulation (Table 3), suggesting adaptations across a wide range of cellular processes. Among the three genes, *hemA* has been previously suggested as a dug target in *Vibrio cholerae* [74].

We did not find any genes identified as experiencing positive selection when we compared the five isolates from horses (Foreground 6, Fig 1) to the rest of the genomes used in the analysis, making it unique across all of our comparisons.

#### Adaptation in Ovis (Foreground 7: Ovis2) and the monophyletic Equi clade (Foreground 8: StraightEqui)

In order to identify genes that under selection in the Ovis and Equi biovars, we compared the genomes from the Ovis clade (Foreground 7, Fig 2) to what we consider to be “Straight Equi” (isolates from buffalo and horses in Foreground 8, Fig 2). We excluded Equi I37 and 162 as they were closer to the Ovis biovars than the other Equi genomes (Fig 1). In this comparison, the Cp1002B genome was used as the anchor for Ovis2 (Foreground 7), and Cp31 for StraightEqui (Foreground 8) (Table 2). Surprisingly, both of these branches shared the same genes undergoing positive selection, the sole exception being a hypothetical protein (Cp31_2281) that was only found to be changing within the Ovis clade (Foreground 7). The fact that both of the clades share the six remaining genes identified as undergoing positive selection indicates that these genes are responding differently to selective pressures that they are experiencing in these environments that these clades are exposed to. These pressures could be different hosts, or something else that we do not yet understand.

Positive selection was identified in sialidase 2 (*nanH*, Cp31_0399), cobaltochelatase subunit CobS (*cobS*, Cp31_1309), lysine exporter protein (*lysE*, Cp31_0893), adhesin 3 (Cp31_0180), and a secreted protein (Cp31_0142). Only Ovis2 had positive selection in Hypothetical protein 4 (Cp31_2281) (Table 3). Sialidase 2 (*nanH*) is also found in *C*. *diphtheria* and *C*. *ulcerans* [86]. Different sialidases in a bacterium can have differences in their substrate specificities and could play important roles in the interaction with other organisms or in the infection of a specific tissue [66]. In *C*. *pseudotuberculosis*, we detected positive selection in 92 sites of sialidase *nanH* and 31 sites in Adhesin 3 (Table 4), suggesting a very active response to whatever the selective pressures are imposing.

### Phylogeny and ecological adaptation

The phylogenetic trees separate biovar Ovis from Equi with at least 90% of confidence value, clearly showing it as a monophyletic group (S2 and S3 Figs). This confirms what has been seen in previous studies [32,33,87]. In addition, the Equi from buffalo and horse formed a clade with two different clusters representing each host. In the phylogenomic trees (S1 and S2 Figs), Equi strain 262 was found to be a sister group of Ovis, as was found in a previous phylogenetic tree using 44 genomes [33]. The *rpoB* gene tree (S3 Fig) shows Equi 262 as the most primitive, but have a similar topology regarding to the other groups. The *rpoB* gene is more efficient at differentiating *Corynebacterium* species than 16S gene [49] and was shown to have power to differentiate biovars and Equi hosts. This tree topologies suggests that Ovis originated from an Equi ancestor, and that the last one is a paraphyletic group [88].

In a previous study, *C*. *pseudotuberculosis* was suggested to be under anagenesis and that Ovis would replace Equi [87]. However, Equi has horse and buffalo as exclusive hosts [19,31] and infections of horses are increasing in frequency in North America [28]. This implies that at least Equi has exclusive hosts in which it would not be outcompeted and replaced by Ovis, and that both biovars (lineages) will probably continue to coexist. Newly divergent lineages can coexist indefinitely when they have exclusive resources [89,90].

Based on our analysis, we feel that *C*. *pseudotuberculosis* evolution fits the “stable ecotype” model of ecological diversification, in which the acquisition of adaptive genes and mutations allows an exploration of a new resource, in this case a new host, creating a new “ecotype” [85,89]. This results in unique selective pressures during the initial expansion by the new clonal population, decreases genetic diversity within the new population by periodic positive selection and genetic drift, and decreases the fitness for the ancestral niche [85,89]. Both populations coexist long enough to accumulate neutral sequence divergence at every locus, being distinguished as multilocus sequence clusters [85,89]. Indeed, Ovis was shown to be i) derived from Equi (this study), ii) more clonal its ancestral biovar [32,33], probably due to decrease in genetic diversity by periodic selection and genetic drift, and iii) to have decreased the fitness for the ancestral niche by losing its capacity to infect horse. The results of our positive selection analysis identified genes under different selective pressures across lineages of *C*. *pseudotuberculosis* that are probably related to changes in ecological niches, which could be represented by expansion into new host ranges.

### False positives for positive selection

The codon models of positive selection analysis are sensitive to data quality. Errors in sequencing, assembly, annotation, alignment and ortholog assignment can lead to false polymorphisms and alignments of non-homologous sites resulting in a statistical signal that is misinterpreted as positive selection [7,91–93]. In this work, five of the total results were identified as false positives (Table 5).

**Table 5:** False positive for positive selection in *Corynebacterium pseudotuberculosis*.

Product	Artifact	Branch	GenBank ID
**Sodium/alanine symporter family protein**	Frameshifts in Ovis and Equi strain 262 (cow)	1: Ovis	Cp1002B_0653
**Zinc ABC transporter, permease protein (*znuB1*)**	Frameshift in Equi strains I37 (cow) and 162 (camel)	1. Ovis	Cp1002B_0053
**HNH endonuclease**	Frameshift in Ovis and Equi MEX30	1. Ovis, 7. Ovis2	Cp1002B_1784
**Lysine exporter protein**	Two frameshifts in Ovis	1. Ovis	Cp1002B_1784

Frameshifts causing alignment of non-homologous codons were identified in proteins mainly related to transport. The false positive found in the Sodium/alanine symporter (Cp1002B_0653) is due to different frameshifts in Ovis and Equi 262, suggesting an independent loss of function, presumably because neither needs this gene for survival.

Frameshift mutations were found in *znuB1* from Equi strains I37 and 262. In fact, the entire *znuB1C1A1* operon of zinc transporter is frameshifted in all the other Equi strains. This operon is in pathogenicity island PiCp2, but another zinc transport operon (*znuB2C2A2*) is found in all *C*. *pseudotuberculosis* strains, which is not located in a pathogenicity island. The loss of function in the zinc transport operon *znuB1C1A1* only in Equi suggests a different selective pressure on this group, with the sequence changes helping it adapt to its particular niche. The loss of specific functionality in specific branches or clades have been suggested as adaptation to different selective pressures in particular niches [94,95]. In bacteria, there is a strong mutational bias toward deleting superfluous sequences by mutation, drift, and selective pressure to reduce the size and redundancy in a genome [90,94].

### Genome variation and the evolution of *C*. *pseudotuberculosis*

Different genome changes involved in host adaptation have been described in bacteria [95,96]. First, the already existent genes can be fine-tuned by positive selection. Second, new genes can be acquired by functional divergence, gene duplication, intragenic recombination or horizontal gene transfer. Third, the genome size can be reduced by loss of sequences due to redundant functions provided by the host, or negative selection [95,96]. Here, we analyzed the positive selection and gene acquisition/loss that could be related to the host preferences of *C*. *pseudotuberculosis*.

In the circular map (Fig 3), there is a gap between PiCp3 and PiCp8 of Cp1002B genome. We examined this region and found an adhesin containing the “Fibrogen-binding domain 1” (RASTtk Cp31_247, GenBank Cp31_2168), flanked by the genes that encode Aspartokinase (*lysC*, Cp31_0184) and Aspartate-semialdehyde dehydrogenase (*asd*, Cp31_0185). Both biovars have this adhesin, but the difference in nucleotide sequence (> 50%) was high enough to be considered a non-homologous sequence by BRIG. The identity between the sequences of the protein in Cp31 and Cp1002B (RASTtk Cp1002B_180, GenBank Cp1002B_184) is 39% with a coverage of 98%. This variation is probably related to adhesion to tissues from different hosts, within the range of each biovar.

Previous studies identified an exclusive sigma factor in PiCp5 of Ovis strains [32,97]. Also, two additional characteristics that differentiate the biovars were recently identified in two other genomic regions using comparative genomics, a Type III restriction-modification system found only in Ovis and a CRISPR-Cas system found only in Equi (Parise *et al*., accepted). Assuming Ovis as a monophyletic clade derived from Equi (S1 to S3 Figs), we checked whether these features are primitive or derived by checking their presence across Equi strains using PATRIC’s Protein Family Sorter [35] and their position in relation to the pathogenicity islands, using GIPSy. The Type III restriction-modification system is in the pathogenicity island PiCp15, which is found only in genomes belonging to Ovis and is absent in all Equi strains. This indicates that PiCp15 was acquired after the separation of Ovis and Equi, presumably by the last common ancestor of all the Ovis isolates.

The CRISPR-Cas genes are in PiCp1 and present in all Equi strains, including strain 262, and one gene is reminiscent in Ovis. This suggests that the CRISPR-Cas genes were acquired by the common ancestor of *C*. *pseudotuberculosis* strains and were lost from the Ovis biovar.

**Fig 4:**
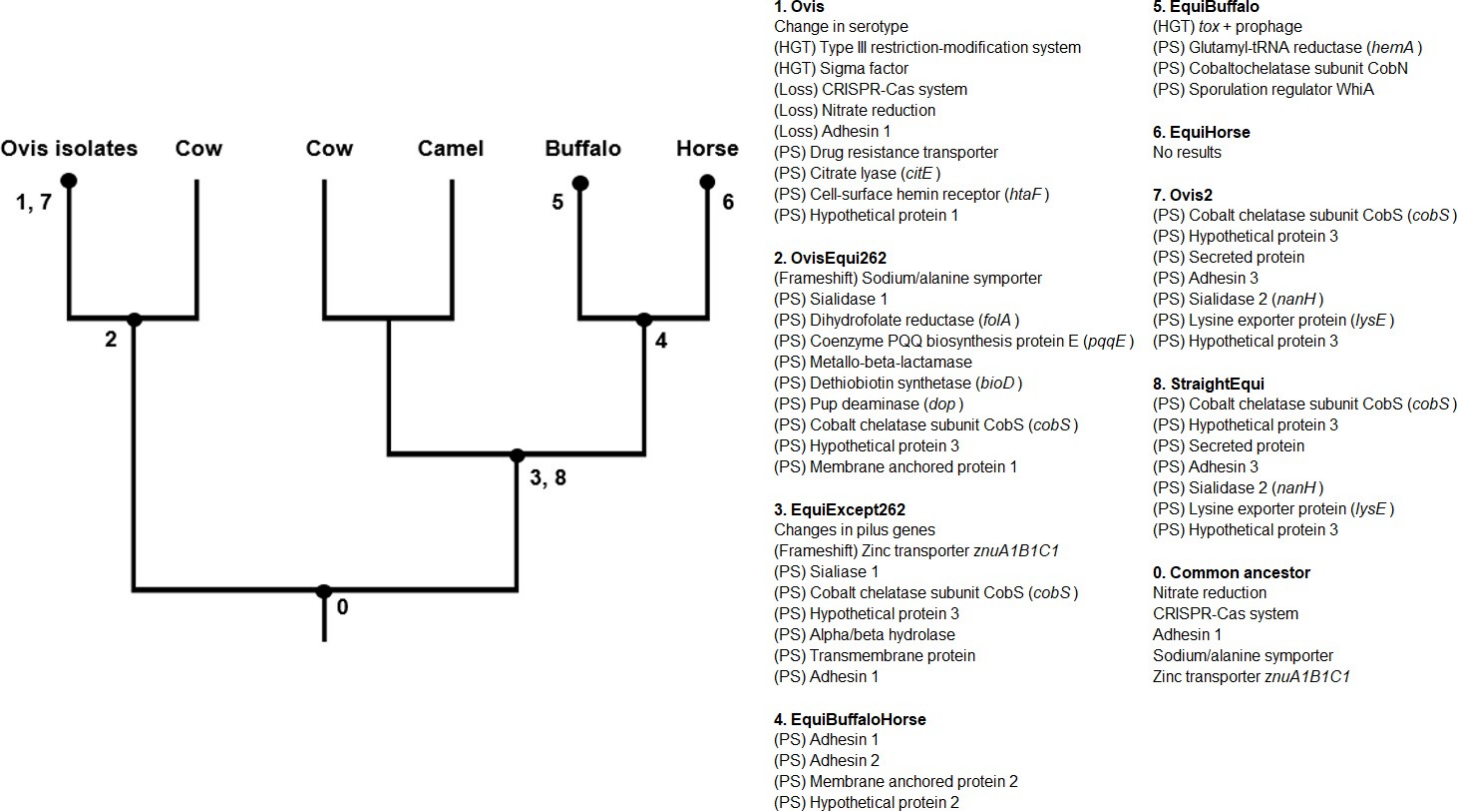
Genome variations in different branches of *Corynebacterium pseudotuberculosis*. HGT–horizontal gene transfer, PS–positive selection.

Various comparative genomics studies have been done in *C*. *pseudotuberculosis* [32,33,84,97–99]. We mapped our data and differences described in previous studies to our phylogenetic tree to clarify the specific changes that have occurred during the evolution and host expansion of this pathogen (Fig 4). In Ovis, previous analyses documented the loss of nitrate reduction related genes [33,76,100], changes in serotype [77], an exclusive Type III restriction-modification system (this study), and a sigma factor in PiCp5 [32,97]. In Equi, previous studies have described frameshifts in pilus genes [32,33] and acquisition of a *tox*+ prophage in PiCp12 [33,101]. Previously, variations in the presence of pathogenicity islands were said to explain most of the phenotypic differences seen between the Ovis and Equi biovars [32]. Here, for the first time, we can see that selective pressures are also occurring, and that they play a likely role in the adaptation of *C*. *pseudotuberculosis* to selective pressures that correspond to the observed differences in phylogeny.

## Conclusions

By performing genome scale positive selection analysis, we have identified what appear to be adaptive mutations in specific genes found in defined phylogenetic clades of *C*. *pseudotuberculosis*. These differences can be seen to correlate with the different hosts that the genomes were isolated from, and with the two biovars described for this species. Many of the proteins identified as being under selection are involved in important processes that are known to increase of survival, including metabolism, cell division, resistance, transport, adhesion. Some of the genes that are under positive selection have previously been identified as potential drug targets in other bacteria, which could indicate a possible future role in treatment or infection prevention. In addition, we have combined a phylogenomic analysis with previously documented changes, and this analysis of positive selection, to show specific changes that have occurred during the evolution of this species. These changes are correlated with both ecological diversification as an expanding host range in this pathogen.

## Supporting information

S1 Fig Phylogenomic tree of *Corynebacterium pseudotuberculosis* generated using the PosiGene pipeline. Equi branches are in blue and Ovis branches are in Orange.
(TIF)

S2 Fig Phylogenomic tree of 29 *Corynebacterium pseudotuberculosis* genomes. Equi branches are in blue and Ovis branches are in Orange. The blue circles represent jackknife values above 90%.
(TIF)

S3 Fig Phylogenetic tree of *Corynebacterium pseudotuberculosis* species tree based on the *rpoB* gene. Equi branches are in blue and Ovis branches are in Orange. The blue circles represent bootstrap values above 90%.
(TIF)

S4 Fig Target groups (foreground branches) 9 to 13 of a *Corynebacterium pseudotuberculosis* phylogeny. The target groups 9 to 12 are subsets of genomes used in target group 1 (Ovis). Target group 13 is a subset of genomes used in target group 5 (EquiBuffalo).
(TIF)

S1 Table Mapping of RASTtk-based and GenBank IDs of each positively selected gene of strain 31 (Equi) and strain 1002B (Ovis). (XLSX)

S2 Table List of positively selected genes and probability of recombination in *Corynebacterium pseudotuberculosis* (*q* < 0.05 for PHI and at least one other test). (XLSX)

S1 File Script used to extract multifasta amino acid files with suitable identifiers from GenBank files. (ZIP)

S2 File Input files used in PosiGene pipeline. (ZIP)

S3 File Individual results of each branch-site analysis. (ZIP)

S4 File Individual results of each branch-site analysis for the genome subsets. (ZIP)
