# Supplementary figures and images for: Rapidly evolving changes and gene loss associated with host switching in Corynebacterium pseudotuberculosis
Source: PLoS One. 2018 Nov 12;13(11):e0207304. doi: 10.1371/journal.pone.0207304 (PMC6231662; doi:10.1371/journal.pone.0207304)

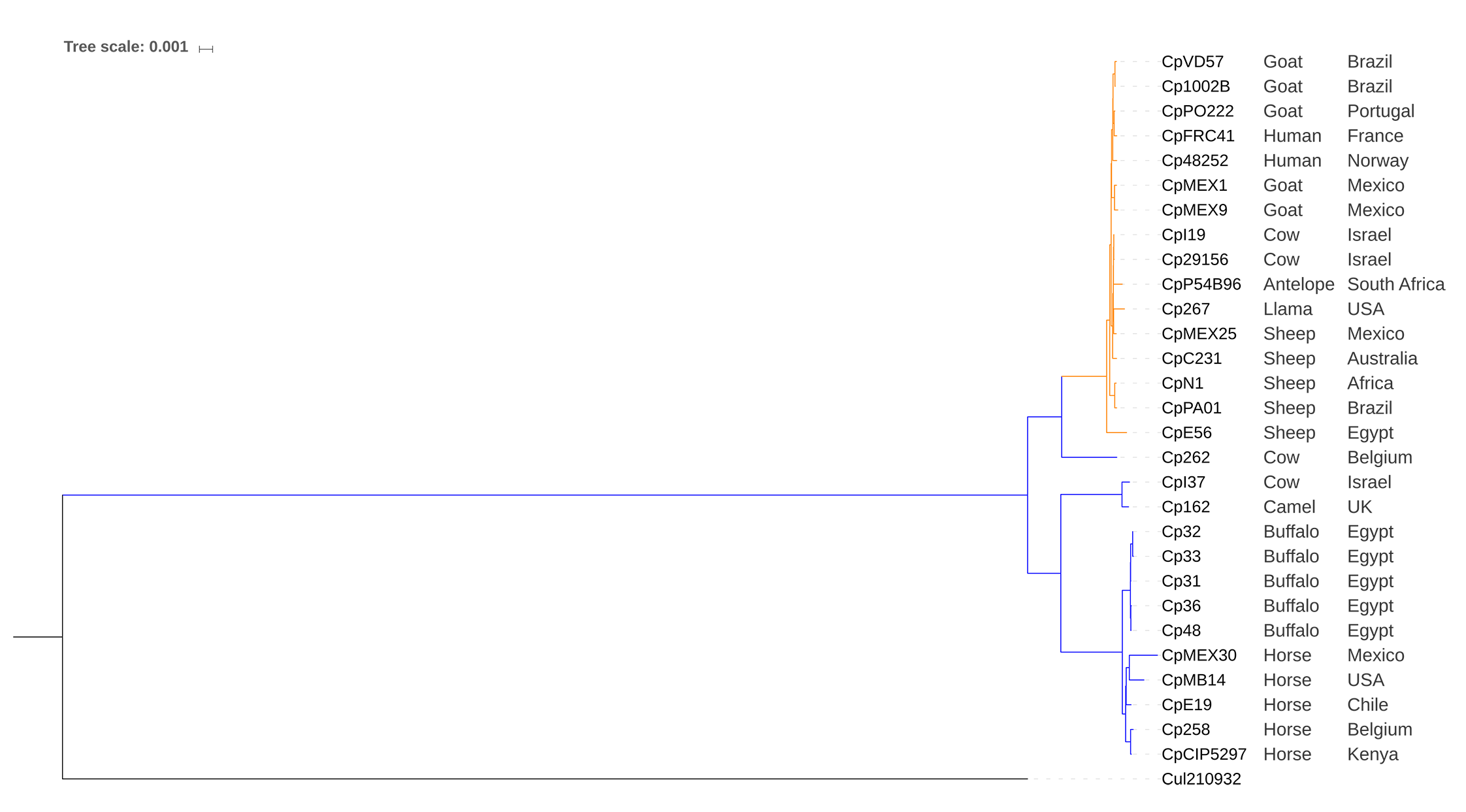

Supplement: S1 Fig — Equi branches are in blue and Ovis branches are in Orange. (TIF) [file pone.0207304.s001.tif]

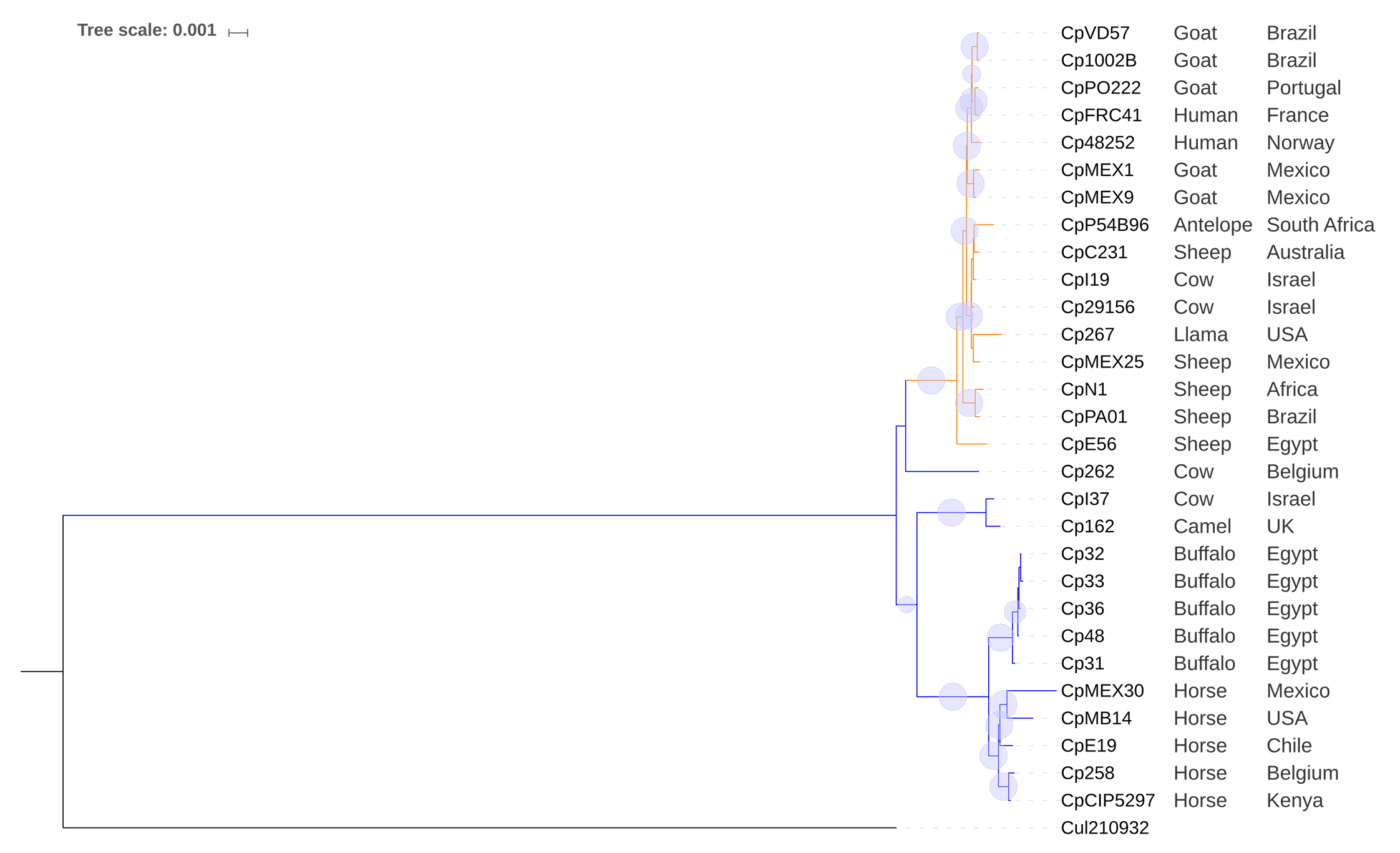

Supplement: S2 Fig — Equi branches are in blue and Ovis branches are in Orange. The blue circles represent jackknife values above 90%. (TIF) [file pone.0207304.s002.tif]

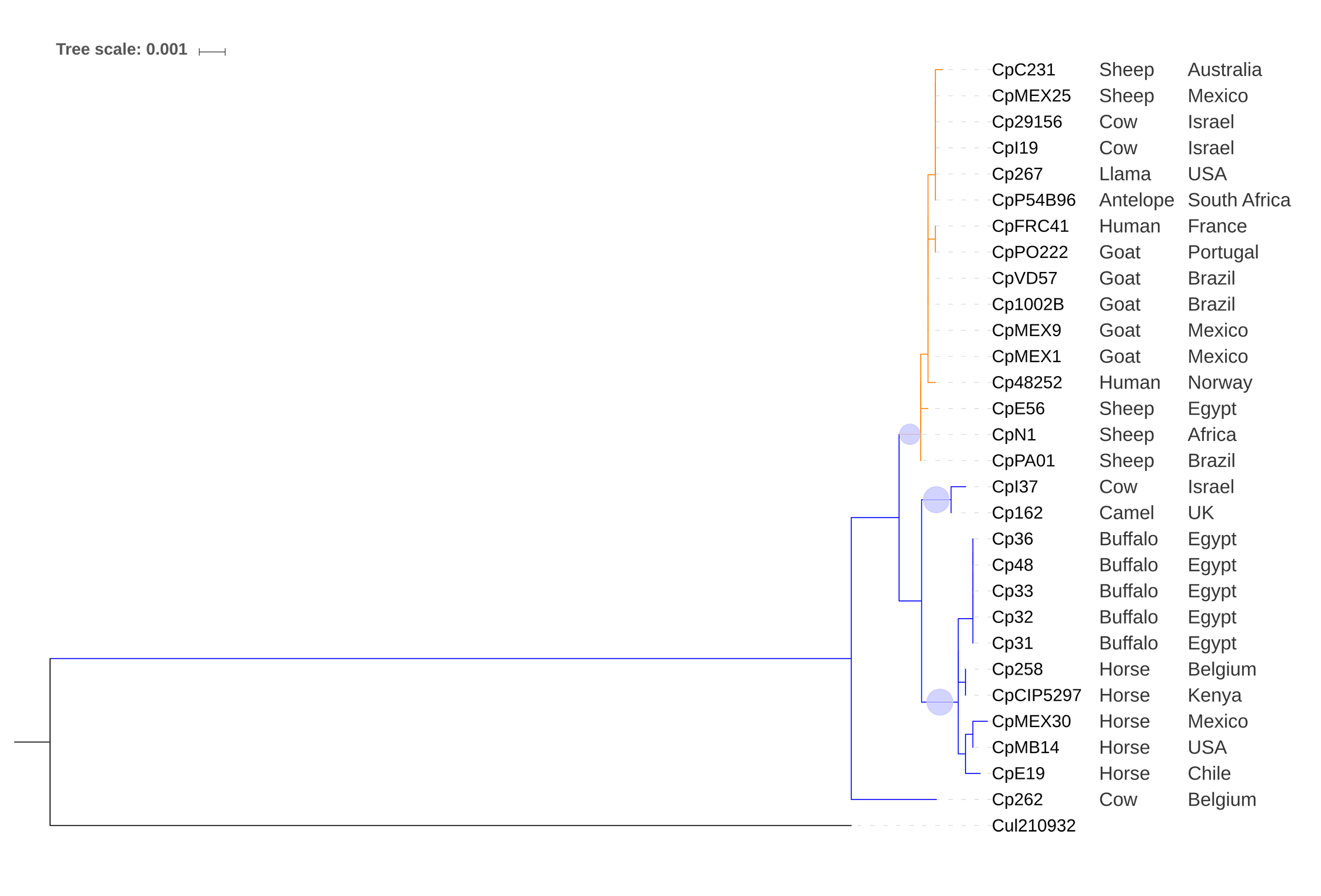

Supplement: S3 Fig — Equi branches are in blue and Ovis branches are in Orange. The blue circles represent bootstrap values above 90%. (TIF) [file pone.0207304.s003.tif]

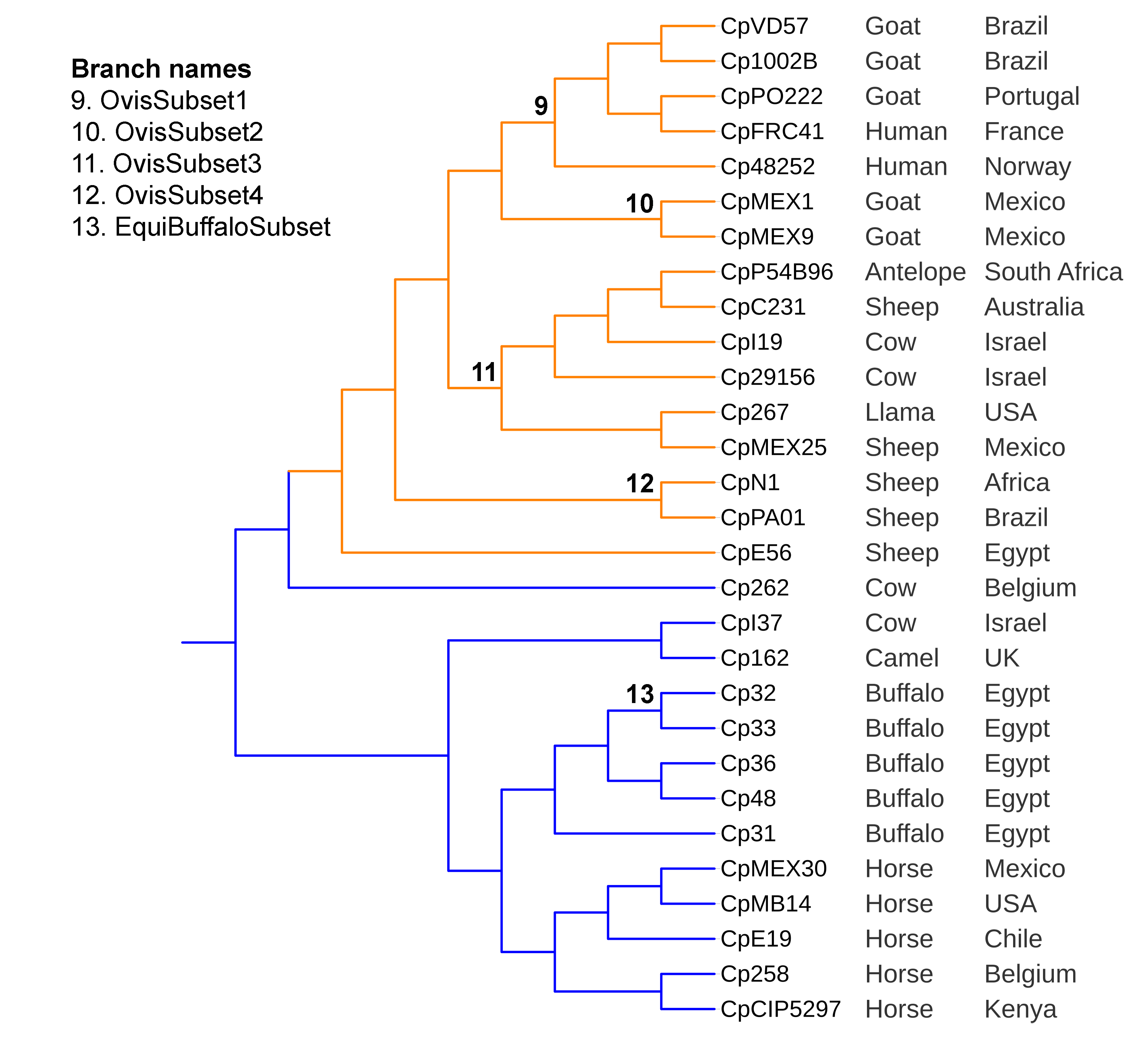

Supplement: S4 Fig — The target groups 9 to 12 are subsets of genomes used in target group 1 (Ovis). Target group 13 is a subset of genomes used in target group 5 (EquiBuffalo). (TIF) [file pone.0207304.s004.tif]
